# High-Quality Draft Genome Sequences of Three Cyanobacteria Isolated from the Limestone Walls of the Old Cathedral of Coimbra, Portugal

**DOI:** 10.1128/MRA.00575-20

**Published:** 2020-06-25

**Authors:** Fabiana Soares, João Trovão, Catarina Coelho, Inês Costa, Nuno Mesquita, Francisco Gil, Lídia Catarino, Susana M. Cardoso, António Portugal, Igor Tiago

**Affiliations:** a University of Coimbra, Centre for Functional Ecology, Department of Life Sciences, Coimbra, Portugal; b University of Coimbra, Center for Physics, Department of Physics, Coimbra, Portugal; c University of Coimbra, Geosciences Center, Department of Earth Sciences, Coimbra, Portugal; d LAQV/REQUIMTE, Chemistry Department, University of Aveiro, Aveiro, Portugal; Portland State University

## Abstract

The recently described species Myxacorys almedinensis and two other cyanobacteria were isolated from the limestone walls of the Old Cathedral of Coimbra, Portugal (UNESCO World Heritage Site). The high-quality genome sequences presented here will be essential for characterization purposes and description of the novel taxa.

## ANNOUNCEMENT

Epilithic and endolithic cyanobacteria are known to cause severe esthetic and physicochemical alterations to stone substrata (1–3). Due to their unique characteristics, photosynthetic nature, and ability to fix nitrogen, they are considered primary colonizers of stone monuments, contributing to future colonization by heterotrophic organisms (4, 5).

During an experimental survey aimed at fully characterizing the community of microalgae and cyanobacteria of the Old Cathedral of Coimbra, Portugal, three novel cyanobacterial taxa were isolated (6). The strain Myxacorys almedinensis coi00094076 (*Synechococcales*) has been recently described (7), whereas studies toward future descriptions of *Nostoc* sp. B (2019) and *Synechococcales* cyanobacterium C are currently being performed. In this article, we present the high-quality draft genome sequences of three isolated strains that were retrieved from the limestone walls of the Old Cathedral of Coimbra. The samples were collected by scraping off green/dark-green biofilms with the help of a sterile scalpel. The cyanobacterial strains were isolated from liquid BG11 enrichment cultures (8) by means of micromanipulation using an inverted microscope and inoculated into flask tubes containing the same culture medium. Inoculates were then incubated at 20 ± 1°C, under a 16:8 h (light-dark) photoperiod (30 to 40 μmol photons m^−2^ s^−1^) until they had developed enough biomass for DNA extraction (6). Genomic DNA was extracted with a DNeasy PowerLyzer PowerSoil kit (Qiagen, USA). Strain identification was performed by molecular analyses of the partial 16S rRNA gene fragments (6). For genome sequencing, libraries were prepared using the Nextera XT library prep workflow (Illumina), and 2 × 150-nucleotide (nt) paired-end reads were generated on an Illumina MiSeq instrument. The genomes were assembled using the programs encompassed in the MetaWRAP pipeline (9), namely, quality trimming was executed using the sliding-window operation in Trim Galore v0.5.0 (10) with default parameters. The final assembly was performed using the SPAdes v3.5.0 (11) assembler with default parameters and k-mer lengths of 21, 33, 55, and 77 nt. The assemblies were subjected to binning with MetaBAT v2.12.1 (12) with default parameters, and a quality check was performed on the final resulting file using CheckM v1.0.12 (13) with default parameters. The complete results regarding the genome sequencing of the three strains are detailed in Table 1.

**TABLE 1:** Detailed data from the genome sequencing of the three strains

Characteristic	Data for strain^*a*^:		
	*Myxacorys almedinensis* A	*Nostoc* sp. B (2019)	*Synechococcales* cyanobacterium C
Strain name according to Soares et al. (6)	*Cyanobacterium* sp. 5	*Cyanobacterium* sp. 1	*Cyanobacterium* sp. 3
No. of reads	907,006	2,466,854	2,556,453
No. of contigs	85	109	79
*N*_50_ (bp)	141,189	228,597	72,306
Genome size (bp)	4,958,574	7,426,239	4,122,371
% G+C content	49.7	41.8	52.0
No. of protein coding sequences	4,367	6,667	4,027
No. of tRNAs	38	88	42
% completeness	99.52	99.55	99.29
% contamination	0.353	0.444	0.471
SRA accession no.	SRX7707471	SRX7707472	SRX7707473
GenBank accession no.	GCA_010091945.1	GCA_010091925.1	GCA_009939295.1

a NCBI strain names.

### Data availability.

The data from this whole-genome sequencing project have been submitted to NCBI under the accession number PRJNA596374. This submission encompasses both the raw data and assembled data.
